# Perceptions and stress of conscience in relation to burnout among nursing staff in older people care settings: a cross sectional study

**DOI:** 10.1186/s12912-023-01529-w

**Published:** 2023-10-13

**Authors:** Shima Nazari, Astrid Norberg, Gunilla Strandberg, Johan Åhlin, Eva Ericson-Lidman, Monir Mazaheri

**Affiliations:** 1 School of Nursing and Midwifery, Tehran University of Medical Sciences, Tehran, Iran; 2 Department of Nursing, Umeå University, Umeå, Sweden; 3 Department of Nursing Science, Sophiahemmet University, Stockholm, Sweden; 4 Department of Neurobiology Care Sciences and Society, Karolinska Institute, Huddinge, Sweden

**Keywords:** Burnout, Nursing staff, Older people care, Perceptions of conscience, Stress of conscience

## Abstract

**Background:**

Considering cultural influences, it is important to study the perceptions and stress of conscience in different contexts. This study aimed to investigate the association between perceptions of conscience, stress of conscience, and burnout among nursing staff working in older people care settings in Tehran.

**Methods:**

This was a descriptive, cross-sectional study. A total of 161 participants completed the Perceptions of Conscience Questionnaire, Stress of Conscience Questionnaire, and Oldenburg Burnout Inventory, 2019. All nursing staff working at the 20 contacted nursing homes agreed to participate in the study. The descriptive and inferential analysis was conducted through SPSS, using T-tests and one-way between-groups analysis of variance, Chi-square and t-tests, Cohen's d (d), Eta-squared (η2), and Phi coefficient (φ), Partial least squares regression (PLSR), jackknife approximate t-tests of the regression coefficients, and jackknife 95% confidence intervals of the regression coefficients.

**Results:**

The nursing staff perceived their conscience mainly as an authority, asset, and warning signal. Impact of workload on family life was the most common source of stress for the nursing staff. Dealing with incompatible demands, the impact of workload on family life, witnessing insulated patients, inability to meet one’s standards in providing care, and perception of conscience as a burden were strongly associated with the burnout.

**Conclusions:**

Perceiving conscience as a warning signal or authority may serve as a buffer against burnout among nursing staff. This study highlights the need for further exploration of perceptions of conscience in different cultural and social backgrounds.

## Background

The concept of conscience has been interpreted differently from theological [1], philosophical [2], psychological [3], and nursing [4] perspectives. Conscience has been described as God’s voice inside human beings in Protestant Christianity and is related to natural law in Catholic tradition [1]. Despite several parallel words expressing conscience in Islam, the word ‘al-damir’ is commonly used, referring to a sense of inner self and pure divine knowledge [5]. Conscience in the nursing literature refers to an inner sense of responsibility reflected in personal and professional integrity [6], which may influence nursing practice ethically [7]. Perceptions of conscience (PC) has been quantitatively investigated among nursing staff (NS) from various backgrounds in primary healthcare centres [8], hospitals [4, 8–12], older people care settings [13, 14], and psychiatric care [13].

In qualitative studies in Iran, nurses described their conscience as a supernatural and inherent sense that helped them provide professional care [15]. They perceived their conscience as moral, a guardian, a director, caring, or a reflection of conscience [16]. The nurses in Iran expressed that professional commitment, responsibility, and religious beliefs may affect their conscience-based care to prevent a troubled conscience [17]. Enrolled nurses (ENs) with an Iranian background, who provided care for older people with Iranian backgrounds in Swedish residential care settings, perceived their conscience as an inner voice that was primarily nurtured by parents and underwent changes throughout their life cycle [18]. NS in Swedish inpatient care considered the warning function of conscience as a motivating factor to provide high-quality care [7]. Conversely, NS in Swedish healthcare settings, who perceived their conscience as a warning signal against hurting others as a burden [19, 20], or unable to follow their conscience at work, reported high levels of stress of conscience (SC) [20].

SC refers to ‘the product of the frequency of ethically problematic situations occurring in healthcare and the perceived degree of troubled conscience as rated by the NS’ [21]. SC has been negatively associated with the quality of older people care [22]. NS with higher levels of SC reported more health complaints than those with lower levels [23]. Lack of time to provide necessary patient care and the influence of workload on family life have been reported as familiar sources of SC among NS in Sweden, Finland, and Australia [8, 24–26]. Feeling obliged to provide insufficient care [9, 24], lowering one’s aspirations for providing good care, the inability to live up to others’ expectations at work [24], and dealing with incompatible demands [24, 26] are other prevalent sources of SC.

In a study in Iran, nurses described their troubled conscience as a reaction to conscience, including guilt, mental preoccupation, discomfort, and fretfulness [27]. ENs with an Iranian background, who worked in Swedish older people care settings, perceived a troubled conscience as living in a state of unease. They were ashamed of admitting having a troubled conscience and reluctant to talk about it because they viewed themselves as uncaring people [28]. NS in Swedish older people care settings indicated that the situations that generated a troubled conscience included being caught between different demands, being pulled away from residents to perform other necessary tasks, being unable to relieve residents’ suffering, and providing insufficient care [29].

PC as a burden [4, 9] and SC were positively associated with burnout [30], which has been reported as a significant problem among NS in care settings in different countries [31]. In a meta-analysis, the overall prevalence of burnout among Iranian nurses was estimated to be 36% [32]. More than 90% of NS at a Norwegian nursing home reported some degree of burden related to ethical problems [33]. In a Swedish older people care setting, 29.7% and 14.3% of NS experienced high burnout associated with emotional exhaustion and depersonalisation, respectively [30]. Burnout has a negative effect on subjective well-being [34], quality of care [35], job satisfaction, and adverse events in older patients [36]. SC and its relationship with burnout among NS in Iran have not yet been studied; however, several studies have reported average to high levels of moral distress [37, 38] and a positive relationship between moral distress and burnout among Iranian NS [38, 39]. The concept of ‘moral distress’ is theoretically connected to the psychological perspective. This is somewhat distinct from SC, which is related to a theoretical-philosophical perspective. However, the concepts of moral distress and troubled conscience have similarities [40].

The majority of the Iranian population is Muslim (10% of Muslim are Sunnis and the remaining are Shi'as). Currently, 6.1% of Iranian people are aged 65 years or older [41]. Most of them live in their own homes; however, the number of nursing home residents continues to rise, especially those with dementia or other restricting conditions [42]. Older people care in Iran has been described to have serious challenges in policymaking, accessibility for all, technical resources, integrity and coordination [43] Iranian nurses have been described to face structural difficulties including nurses shortage and inappropriate ward environment as well as lack of training in communication with older people and care ethics in older people care settings [44].

Considering the influence of culture on PC and SC, it is essential to improve our understanding of these concepts in culturally diverse populations. However, we could not find any study on burnout and its association with PC and SC in NS treating Iranian older adults in care settings. Furthermore, most quantitative studies on SC are geographically limited to Scandinavian countries as the SC questionnaire was developed in Sweden [21]. This calls for further investigation in diverse sociocultural contexts and other countries. This study thus aimed to investigate the association between PC and SC and burnout among nursing staff working in care settings for older people in Tehran.

## Methods

### Research design

A descriptive cross-sectional study was conducted between April and June 2019.

### Setting

The participants were recruited from 20 nursing homes and one geriatric hospital ward located in different areas of Tehran Province, Iran.

### Data collection

Following permission from the social welfare organisation, the first author contacted nursing home managers and the geriatric ward head nurse to recruit NS providing care for older people. The NS included registered nurses (RNs), ENs, and nursing assistants (NAs).

In Iran, RNs have at least a four-year bachelor’s degree in nursing science, ENs pass a two-year academic program, and NAs pass a short formal care training course [45]. NS provide care for older people with multiple comorbidities, including dementia and physical disabilities, during the day (7 am to 3 pm) or evening/night shift (3 pm to 7 am).

Three RNs (including one head nurse) and two NAs on the day shift and two RNs and two NAs on the evening/night shift provided care to 11 residents in the hospital geriatric ward. Care for older people in Iranian nursing homes involves teamwork, usually managed by a general practitioner. Therefore, staffing patterns differ depending on the residents’ needs. Based on Iranian social welfare guidelines, one RN and one EN on the day shift and two ENs on the evening/night shift were responsible for approximately 50 residents. One NA was assigned to every seven dependent residents (Barthel index 0–49) or ten independent residents (Barthel index 75–99) [46]. Weekends have lower staffing.

All NS who were contacted, including RNs, ENs, and NAs, with at least one year of experience caring for older adults agreed to participate in the study.

Data were collected using four different questionnaires: (1) sociodemographic, (2) the revised Perceptions of Conscience Questionnaire (PCQ) [8], the revised Stress of Conscience Questionnaire (SCQ) [8], and the Oldenburg Burnout Inventory (OLBI) [47]. Appointments were made with each participant to hand in the questionnaires and answer any questions if needed. All distributed questionnaires were collected by the research team, which resulted in no dropout cases.

### Instruments

The three published questionnaires were translated from English into Persian by a professional translator. They were then back-translated into English using English-speaking RNs. Qualitative, face, and content validity were determined according to the method described by Polit and Beck [48] by a panel of experts, who assessed the content validity ratio (CVR), item-content validity index (I-CVI), and scale-content validity index-average (S-CVI-Ave). Finally, linguistic validation was performed by the first author and the professional translator to ensure that the phrases of the questionnaires could support the interpretation of the findings.

The sociodemographic questionnaire included age, sex, marital status, education, clinical experience, and care experience of older people.

The revised PCQ consisted of 16 items. NS assessed their PC (its origin, function, and significance) on a 6-point Likert-type scale including ‘1 = no, totally disagree’, 2 = no, disagree somewhat, 3 = Yes, agree somewhat 4 = yes, agree somewhat, 5 = yes, mainly agree, and ‘6 = yes, entirely agree’. According to a factor analysis of the PCQ, Åhlin et al. [8] reported that NS perceived conscience as an authority (items 6,10,14,15), an asset (items 7,8), a warning signal (items 3,4,5), demanding sensitivity (items 1,2,10), or a burden (items 11,12,16) and dependent on culture (items 9,13). Different studies have been conducted using the PCQ [10–12]. In our study, Cronbach’s α for the PCQ was estimated to be 0.87. The revised SCQ consists of nine two-part items, including A and B questions that measure stress originating from a troubled conscience based on staff experience in healthcare. The A questions assessed the frequency of certain difficult ethical situations on a 6-point Likert-type scale from ‘0 = never’ to ‘5 = everyday’. The following B questions estimated the amount of troubled conscience related to each given situation on a 10 cm visual analogue scale from ‘0 = no, it gives me no troubled conscience at all’ to ‘5 = yes, it gives me a very troubled conscience’. The SCQ index for each item was calculated by multiplying the score from each A question by the score of its analogous B question. The SCQ index ranged from 0 to 25 points for each item. This questionnaire has been used in different countries [24, 25, 49]. In the present study, Cronbach’s α for the SCQ was estimated to be 0.82.

The OLBI has two dimensions: exhaustion (affective, physical, and cognitive) and disengagement. Each subscale consists of eight items, four of which are positively and four of which are negatively worded. These were presented in a mixed order with four response alternatives, ranging from ‘1 = totally disagree’ to ‘4 = totally agree’ [47]. In the present study, the Cronbach’s α of the OLBI was 0.85.

### Statistical procedures

The analysis was conducted using SPSS version 25 for Windows (SPSS, Chicago, IL, USA) and R software (version 3.5.1, 2018–07-02). No replacement of missing values was performed since missing values were low and missing at random. Furthermore, missing values for the PCQ ranged from 0% to 5.6% and 0% to 0.6% for the OLBI. Cronbach’s alphas for the total scales were 0.685 for the SCQ, 0.872 for the PCQ 0.852, 0.757 for the burnout dimension exhaustion (EX) and 0.758 Disengagement (DIS).

T-tests and one-way between-groups analysis of variance were performed to investigate differences in SC and the two burnout dimensions depending on demographic variables. Chi-square and t-tests were used to examine associations and differences in dichotomised items from the PCQ concerning demographic variables. Effects were also analysed using the following effect size measures: Cohen's d (d), Eta-squared (η2), and Phi coefficient (φ). The criteria set by Cohen (1988) regarding effect sizes were used: small (d ≥ 0.2, η2 ≥ 0.02, φ ≥ 0.10), moderate (d ≥ 0.50, η2 ≥ 0.06, φ ≥ 0.30), and large effects (d ≥ 0.80, η2 ≥ 0.14, φ ≥ 0.50).

Partial least squares regression (PLSR) was used to identify items of importance in the SCQ and PCQ in relation to the two OLBI subscales of exhaustion and disengagement. The jackknife approximate t-tests of the regression coefficients were used to evaluate the independent importance of each item in the SCQ and PCQ to explain the variation in the response variables. Statistical significance was set at *p* < 0.05. The jackknife 95% confidence intervals of the regression coefficients were used in the same manner to facilitate graphical interpretations of the findings. The number of components in the PLSR model was selected by examining the various validation plots.

### Ethical considerations

This study was approved by the Organizational Joint Committee on Ethics in Biomedical Research, Tehran University of Medical Sciences (IR.TUMS.FNM.REC.2018–067). Participants were given verbal and written information about the study. They were assured that their participation was voluntary and that they could withdraw at any time without any explanation or consequences. All questionnaires were completed without noting the participants’ names or contact details, organizational characteristics, or any additional information that could violate their confidentiality. All the data were coded as they were entered into the datasheets. Printed copies of the questionnaires were saved in a locked cabinet at the first author’s workplace where no one else had access. All methods were carried out in accordance with relevant guidelines and regulations.

## Results

### The participants

In total, 161 NS, including 43 RNs/ENs and 118 NAs, participated in this study. The participants included 149 NS from nursing homes and 12 from geriatric wards. Participants were 19 to 64 years old (36.7 ± 9.3). Most were women (75.8%), married or cohabiting (51.6%), and had a high school diploma (49.1%). Approximately 42.2% of the participants had less than five years of clinical experience, and 49.7% had less than five years of experience in caring for older people. Participants’ demographics are presented in Table 1.

**Table 1:** Background data of the nursing staff working in older people care (*N* = 161)

**Gender**	
Male	39 (24.2%)
Female	122 (75.8%)
**Age**	
Mean ± Sd	36.7 ± 9.3
**Clinical Experience**	
Less than 5 years	68 (42.2%)
5 to 10 years	45 (28.0%)
10 to 15 years	25 (15.5%)
More than 15 years	23 (14.3%)
**Clinical experience in caring of older people**	
Less than 5 years	80 (49.7%)
5 to 10 years	43 (26.7%)
10 to 15 years	22 (13.7%)
More than 15 years	16 (9.9%)
**Education**	
Finishing elementary school (Cycle in Iran)	39 (24.2%)
High school diploma (Completing high school)	79 (49.1%)
Degree in nursing (Academic education)	43 (26.1%)
**Marital status**	
Married	83 (51.6%)
Never married	45 (28.0%)
Divorced	26 (16.1%)
Widow	7 (4.3%)

### Perceptions of conscience, stress of conscience, and burnout

The majority of participants agreed with the following statements: ‘We should follow our conscience, no matter what other people think’ (PCQ6) (89.5%), ‘When I follow my conscience, I develop as a human being’ (PCQ15) (89.4%), ‘I follow my conscience at work’ (PCQ8) (88.5%), ‘God speaks to us through our conscience’ (PCQ14) (87%), ‘Our conscience warns us against hurting others’ (PCQ5) (86.6%), and ‘Our conscience expresses our social values’ (85.4%). Most (71.9%) of the NS disagreed with the statement, ‘I have to deaden my conscience to keep working in healthcare’. About 71.3% perceived their conscience as far too strict. Further details are provided in Table 2.

**Table 2:** A descriptive analysis of PCQ items of the nursing staff working in older people care

Item no	Question	% responses for each score. range 1–6 1 2 3 4 5 6	% agree/not agree
PCQ 1	The voice of conscience must be interpreted (Demanding sensitivity)	9.5 10.8 5.7 19.0 28.5 26.6	74.1/25.9
PCQ 2	You need an inner peace to be able to hear the voice of conscience (Demanding sensitivity)	8.8 6.3 7.5 12.5 32.5 32.5	77.5/22.5
PCQ 3	We cannot avoid the voice of conscience (Warning signal)	11.3 4.4 3.8 10.7 30.8 39.0	80.5/19.5
PCQ 4	Our conscience warns us against hurting ourselves (Warning signal)	10.7 6.3 6.9 9.4 37.7 28.9	76.1/23.9
PCQ 5	Our conscience warns us against hurting others (Warning signal)	5.7 6.4 1.3 8.3 38.2 40.1	86.6/13.4
PCQ 6	We should follow our conscience no matter what other people think (Authority)	3.9 3.9 2.6 9.2 33.6 46.7	89.5/10.5
PCQ 7	In my workplace I can express what my conscience tells me (Asset)	5.6 6.2 5.6 15.5 43.5 23.6	82.6/17.4
PCQ 8	I follow my conscience in my work (Asset)	1.9 6.4 3.2 12.2 35.3 41.0	88.5/11.5
PCQ 9	Our conscience can give us wrong signals (Depending on culture)	21.1 19.7 7.2 16.4 19.1 16.4	52.0/48.0
PCQ 10	Our conscience fades away if we do not listen to it (Authority & Demanding sensitivity)	9.6 9.6 1.9 16.6 26.8 35.7	79.0/21.0
PCQ 11	I have to deaden my conscience in order to keep working in healthcare (Burden)	28.1 35.0 8.8 6.9 13.1 8.1	28.1/71.9
PCQ 12	My conscience is far too strict (Burden)	8.9 9.6 10.2 21.0 28.7 21.7	71.3/28.7
PCQ 13	Our conscience expresses our social values (Depending on culture)	1.9 7.6 5.1 19.7 38.2 27.4	85.4/14.6
PCQ 14	God speaks through our conscience (Authority)	5.6 2.5 5.2 21.7 26.1 39.1	87.0/13.0
PCQ 15	When I follow my conscience, I develop as a human being (Authority)	0.6 3.1 6.8 12.4 35.4 41.6	89.4/10.6
PCQ 16	When I cannot live up to the standards, I set for myself, I get a troubled conscience (Burden)	5.0 6.2 5.0 16.8 36.6 30.4	83.9/16.1

The item, ‘Is your work in healthcare ever so demanding that you do not have the energy to devote yourself to your family as you would like?’ (SCQ7, m = 9.03) had the highest SCQ index score. The following items are presented in descending order of mean scores: ‘Do you ever see patients being insulted and/or injured?’ (SCQ4, m = 6.72) and ‘Do you ever have to deal with incompatible demands in your work?’ (SCQ3, m = 6.39). The item, ‘Are you ever forced to provide care that feels wrong?’ (SCQ2, m = 2.45) had the lowest SCQ index score. Details are presented in Table 3. The total mean SCQ index was 46.27 ± 41.45.

**Table 3:** Stress of Conscience Questionnaire (SCQ) items:—Mean values and 95% confidence intervals for each item in the SCQ

Item no	Question	Mean (95% CI)
SCQ 1	How often do you lack time to provide the care the patient needs?	4.67 (3.52–5.82)
SCQ 2	Are you ever forced to provide care that feels wrong?	2.45 (1.69–2.81
SCQ 3	Do you ever have to deal with incompatible demands in your work?	6.39 (5.14–7.63)
SCQ 4	Do you ever see patients being insulted and/or injured?	6.72 (4.14–9.30)
SCQ 5	Do you ever find yourself avoiding patients or family members who need help or support?	2.97 (2.07–3.87)
SCQ 6	Is your private life ever so demanding that you do not have the energy to devote yourself to your work, as you would like?	4.12 (3.15–5.09)
SCQ 7	Is your work in healthcare ever so demanding that you do not have the energy to devote yourself to your family, as you would like?	9.03 (7.60–10.46)
SCQ 8	Do you ever feel that you cannot live up to others’ expectations of your work?	4.45 (3.45–5.45)
SCQ 9	Do you ever lower your aspirations to provide good care?	5.68 (4.41–6.95)

Mean burnout scores for NS were calculated as exhaustion (19.96 ± 4.83) and disengagement (19.16 ± 5.06).

### Relationship with demographic factors

A higher proportion of women (76.9%, *n* = 93) agreed with the statement, ‘My conscience is far too strict’ (PCQ12) than men (52.8%, *p* = 0.005, φ = 0.224). There was a significant difference in age between NS that agreed, compared with those that did not agree, with the statements ‘We cannot avoid the voice of conscience’ (PCQ3) (m = 37.6 vs 33.2, *p* = 0.015, d = 0.49) and ‘I follow my conscience in my work’ (PCQ8) (m = 37.7 vs 32.1, *p* = 0.014, d = 0.64).

Women reported significantly (*p* = 0.009) higher levels of SC (m = 10.1) in response to the item ‘Is your work in healthcare ever so demanding that you do not have the energy to devote yourself to your family as you would like?’ (SCQ7) than men did (m = 5.7, d = 0.50). One-way between-group analysis of variance showed statistically significant differences in responses to SCQ 3, 5, 6, and 9, and the SCQ index total depended on educational background. Post-hoc analyses revealed that NS who had finished high school (m = 7.9) or had a bachelor’s degree (m = 7.2) reported significantly higher levels of SC in response to ‘Do you ever have to deal with incompatible demands in your work?’ (SCQ3) compared to those who had finished elementary school (m = 2.7, η2 = 0.08).

NS who had finished high school (m = 4.1) assessed significantly higher degrees of SC from avoiding patients or family members who needed help or support (SCQ5) compared to those who had finished elementary school (m = 1.0, η2 = 0.05). In addition, those who had finished high school (m = 56.38) or had a bachelor’s degree (m = 47.16) reported significantly higher degrees of SC on the total SCQ index than those who had finished elementary school (m = 23.84, η2 = 0.11).

### Associations between the SCQ and PCQ in relation to exhaustion and disengagement

**Fig. 1:**
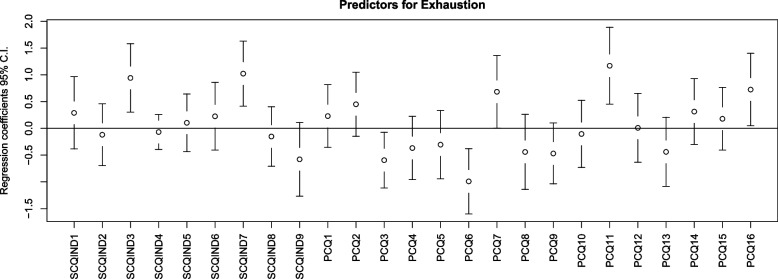
Predictors for exhaustion

The PLSR model with exhaustion as the response variable showed that the items (i.e. predictors) of ‘Do you ever have to deal with incompatible demands in your work?’ (SCQ3, *p* = 0.004); ‘Is your work in healthcare ever so demanding that you do not have the energy to devote yourself to your family, as you would like?’ (SCQ7, *p* = 0.001), ‘At my workplace, I can express what my conscience tells me’ (PCQ7,*p* = 0.049), ‘I have to deaden my conscience to keep working in healthcare’ (PCQ11,*p* = 0.001), and ‘When I cannot live up to the standards, I set for myself, I get a troubled conscience’ (PCQ16,*p* = 0.03) were positively associated with exhaustion. Items of ‘We cannot avoid the voice of conscience’ (PCQ3, *p* = 0.026) and ‘We should follow our conscience, no matter what other people think’ (PCQ6, *p* = 0.001) were negatively associated with exhaustion (Fig. 1). The PLSR model consisted of three components, and the variables explained 41.88% of the variance in response variable, namely exhaustion.

**Fig. 2:**
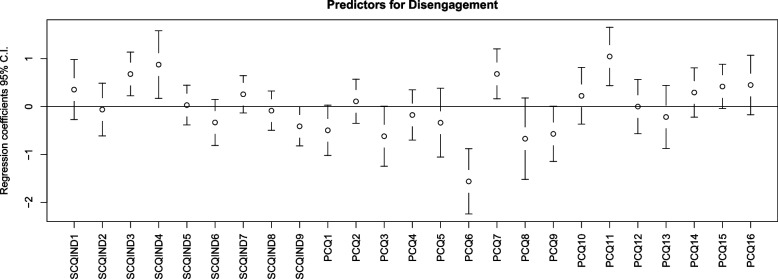
Predictors for disengagement

‘Do you ever have to deal with incompatible demands in your work?’ (SCQ3, *p* = 0.003); ‘Do you ever see patients being insulted and/or injured?’ (SCQ4, *p* = 0.016), ‘At my workplace, I can express what my conscience tells me’ (PCQ7, *p* = 0.011), and ‘I have to deaden my conscience to keep working in healthcare’ (PCQ11, *p* < 0.001) were positively associated with disengagement. Furthermore, there was a negative association between ‘We should follow our conscience, no matter what other people think’ (PCQ6, *p* < 0.001) and disengagement (Fig. 2). The PLSR model consisted of three components, and the variables explained 42.65% of the variance in the response variable, namely disengagement.

## Discussion

This study explored PC and SC and their associations with burnout among NS in older people's care settings in Tehran. Despite some differences, there are similarities between the findings of our study and those of studies conducted in healthcare settings in other countries.

In the current study, NS perceived conscience as an authority, an asset, and a warning signal. About 28.1% of them reported that they had to deaden their conscience to keep working, while 76.9% of female NS considered their conscience far too strict.

The majority of the Iranian population is Muslim; consequently, the culture of Iran can be mostly described by Islamic values and principles. From an Islamic viewpoint, conscience is a divine force inside human beings that warns them against wrong actions. Subsequently, people finally reach the ultimate degree of perfection and salvation [50]. Therefore, most Muslims believe that conscience should be incontestably followed. As an authority, PC can be related to a person’s commitment to a pivotal individual or social value that forms human identity; hence, the main function of PC as an authority is to protect people from hurting themselves and others [4]. The warning function of conscience can serve as a guide against wrongdoing. That is, conscience requires a person to be sensitive to and follow it, regardless of what others think. Consequently, conscience can be perceived as an asset that, by following it, can help a person evolve into a human being. On the contrary, having too strict a PC may raise questions about the validity of its performance [19].

In our study, NS mostly agreed that God speaks to them through their conscience and that following their conscience could help them develop into human beings which is different with the Swedish studies. ENs with an Iranian background, who worked in a Swedish people older care setting, perceived their conscience as an inner guide formed by an upbringing that should be followed at work to be a good person. Although they did not perceive themselves as religious persons and were educated in the Swedish system, their viewpoints could have been influenced by their religious background [18]. In qualitative studies in Iran, NS described nursing as a spiritual profession and a divine blessing. They believed that spirituality is a kind of internal commitment related to God or conscience [51] and that religious beliefs impact their conscience-based care [17]. In Swedish, Turkish, and Iranian studies, NS considered their conscience as a voice that must be interpreted rather than God’s voice to follow straightaway [10, 12, 52] which is different with our study findings. Åhlin et al. [8] reported that the high rate of missing values for the item addressing ‘whether God speaks to us through our conscience’ may be due to the large percentage of Swedish people who recognise themselves as non-believers in God [53]. They concluded that this question was probably of greater relevance among believers in God [8]. However, variations in the findings of studies conducted in Islamic countries, such as Iran [10] and Turkey [12], show a need for further studies in diverse contexts and practices when interpreting one’s PC. Notably, the religious beliefs of participants in these studies were not reported.

Compared to men, women in our study experienced higher levels of SC because of the influence of workload on their family life. NS, who are women in other care settings, experienced higher levels of SC compared to men due to lack of time, being forced to provide insufficient care [9, 20], having to deal with contradictory demands, receiving less support from their colleagues, and having fewer opportunities to talk about work-related problems with their colleagues [9]. However, a study in China showed that married but childless NS experienced the highest level of SC, compared to married NS who had children, and concluded that life changes after childbirth might divert one’s attention from work and reshape one’s feelings and perceptions of difficult situations in work [49]. Work–family conflict can occur in two directions: (1) when demands and responsibilities from one’s role (usually at home) interferes with another role in another domain (usually at work) and (2) when work life is incompatible with family life [54]. Being a woman has been shown to have an increased risk of being exposed to work-family conflict [55], which is more common among working mothers than among childless women [56]. Motherhood and housekeeping are still considered the main responsibilities of Iranian women, even those who are employed [57]. It seems that having greater responsibility for home and family makes women more prone to experience a higher degree of SC due to workload influences on their family lives.

In this study, NS with higher levels of education reported a greater degree of total SC regarding confronting contradictory demands at work, avoiding helping patients or their families, the influence of family life on their work, and lowering their aspirations for good care. RNs in other care settings experienced higher SC than NAs due to multifaceted and higher education, a deeper understanding of good care for older people, being leaders of nursing teams [24], avoiding patients and their families, and falling short of expectations [14]. It has been shown that gerontology education can increase the knowledge and attitudes of NS regarding older people's care [58, 59]. Furthermore, the inability to provide the desired level of care can lead to feelings of SC among NS [24].

In the present study, the PLSR showed that NS with high levels of SC (SCQ3,7) and PC as a burden (PCQ11,16) suffered from high levels of exhaustion, while those with high levels of SC (SCQ3,4) and PC as a burden (PCQ11) showed high levels of disengagement. The positive associations with burnout and an inability to live up to one’s standards in providing good care [9, 57], lack of time, workload [52, 60], inability to live up to others’ expectations at work, inability to express conscience at work [52], and PC as a burden have been documented in other studies, which primarily used the Maslach Burnout Inventory [9, 52, 60]. Even if factorial and convergent validity between the OLBI and MBI is satisfactory [61], comparisons with other studies are difficult because different questionnaires were used. Regardless, there were similarities; for example, NS working in care settings for older people had common burdensome experiences. Most of the NS in the current study perceived their conscience as too strict and nearly 1/3 of them reported that they had to deaden their conscience to keep working. Studies have shown that having to deaden one’s conscience [9, 52, 60] and considering one’s conscience too strict are significantly related to burnout [9, 13, 19, 21]. Nurses described conscience as the driving force for providing high-quality care and a source of sensitivity for patients and their families’ expectations and needs [7]. The deadening of one’s conscience might be an obstacle to their ability to live up to their standards in providing good care [9]. Deadening one’s conscience could be related to the ability to collaborate with colleagues at work and maintain a personal identity as a ‘good caregiver’ [18]. In addition, perceiving one’s conscience as too strict can lead to doubts about the authenticity of its function in guiding individual actions [19].

The influence of workload on family life has been reported as one of the main sources of burnout in NS [52]. Significant associations have been observed between work-family conflict, burnout, and work-related stress [62]. Greater home and family responsibilities for women increase their likelihood of experiencing stress owing to the influence of workload on family life; thus, leading to burnout. Since women usually constitute a larger proportion of NS, it is important to adjust their work pressure to prevent burnout and other negative health outcomes.

According to the current study, PC as a warning signal and authority might serve as a buffer against burnout. It has been shown that PC as a guide and an asset that cannot be avoided could help prevent NS burnout [13]; however, Swedish NS have stated that the conscience can sometimes emit wrong signals [4], which can lead to SC [19, 20] and burnout [14]. It appears that PC as a warning signal has a dual nature. On the one hand, by bringing awareness to action as right or wrong, the warning function could be considered a guide. Comparatively, if the signals are incorrectly interpreted, it could lead to SC and burnout. Further qualitative and quantitative investigations regarding different backgrounds are needed to better understand the variations in PC, SC, and burnout among NS in older people care settings.

A limitation of the study is that the researchers met the NS of the included nursing homes in person to ask them to participate in the study and to hand-in the questionnaire later. This might have impact on their freedom of choice to participate in the study. However, all the participants were clearly informed about the participation was totally voluntary and they could withdraw at any time without needing to explain it or any consequences.

Investigating a subject of interest through quantitative approaches and methods, would limit our in-depth understanding of phenomenon. We suggest further exploring of perception of conscience and stress of conscience with help of qualitative approaches. Moreover, the study needs to be replicated in other cultural contexts including others with Islamic background to assess the similarities and differences.

## Conclusions

Understanding the correlation between SC and PC as a burden and burnout rate is necessary to tailor NS working conditions. In this study, being exposed to contradictory demands, the influence of workload on family life, being a witness to patient insulation, the inability to meet one’s standards, and PC as a burden were strongly related to burnout among NS in older people care settings in Tehran. Moreover, the influence of workload on family life, higher educational level, and being a woman was related to a higher degree of SC. Women also perceived their conscience far too strict more than men. NS mostly perceived their conscience as a warning signal, authority, or asset. In this regard, acknowledging the warning function of conscience and following it, regardless of others’ opinions, can protect NS from burnout. Overall, this study’s findings emphasized that NS who are women are more prone to experiencing SC and burnout at work; thus, these findings can be used to develop tailored interventions for NS to reduce the probability of burnout.

## Data Availability

The datasets generated and/or analysed during the current study are not publicly available due to local ethical guidelines but are available from the corresponding author on reasonable request.

## Abbreviations

CVR: Content validity ratio
ENs: Enrolled nurses
I-CVI: Item-content validity index
NAs: Nursing assistant
RNs: Registered nurse
OLBI: Oldenburg Burnout Inventory
PC: Perception of conscience
PCQ: Perceptions of Conscience Questionnaire
PLSR: Partial Least Square Regression
SC: Stress of conscience
SCQ: Stress of Conscience Questionnaire
S-CVI-Ave: Scale-content validity index-average
φ: Phi coefficient
